# Expert goalkeepers’ and coaches’ views on anticipation and cue utilisation facing backcourt throws in handball goalkeeping

**DOI:** 10.3389/fspor.2023.1215696

**Published:** 2023-10-09

**Authors:** Kim Huesmann, Jörg Schorer, Dirk Büsch, Jelto Witt, Florian Loffing

**Affiliations:** ^1^ Department of Movement Science, Institute of Sport Science, University of Oldenburg, Oldenburg, Germany; ^2^ Department of Training Science, Institute of Sport Science, University of Oldenburg, Oldenburg, Germany; ^3^ Department of Performance Psychology, Psychological Institute, German Sports University, Cologne, Germany

**Keywords:** team sport, expertise, interception, qualitative research, perceptual-cognitive skill, coaching

## Abstract

**Introduction:**

Handball goalkeepers have to act under severe spatio-temporal pressure in both standardised (e.g., 7 m penalty) and non-standardised situations (e.g., backcourt throws) which require them to predict action outcome before ball flight is visible. So far, research on goalkeepers’ cue utilisation for anticipation of an opponent’s action has mainly focused on 7 m throw situations whereas little attention has been paid to the latter, more complex and far more frequently occurring backcourt throw situations.

**Methods:**

To address this gap, we conducted semi-structured interviews with *N* = 6 expert handball goalkeepers and goalkeeper coaches [all of whom were (former) expert handball goalkeepers] on anticipation and cue utilisation when facing backcourt throws. The interviews were subsequently transcribed, coded and results were inductively as well as deductively categorised by means of a thematic analysis.

**Results:**

Results reveal a variety of kinematic and contextual cues relevant for action anticipation that become available before the game and before or during the throw. Participants reported to use information from the offence (e.g., thrower’s jump; opposing team’s task distribution) and the defence (e.g., defensive players’ strategies, block position) for anticipation in backcourt throw situations. Additionally, we identified several factors that influence cue availability and utilisation.

**Discussion:**

Our findings provide a thorough basis to (a) guide future research that yields questions on kinematic and contextual cue integration and in-situ cue usage as well as (b) inform the development of training programs to foster goalkeepers’ anticipatory skill.

## 1. Introduction

Goalkeepers, across sports, hold a unique position within a team. They are usually the last instance between the ball and the goal which requires them to be at their highest performance level and avoid mistakes at all times to successfully prevent the opposing team from scoring a goal. One of the crucial factors of goalkeeper performance is the ability to perform under high spatio-temporal pressure. Handball goalkeepers for instance are left with very little time to react to an opponent’s 7 m penalty throw. Less standardised situations such as backcourt throws, which occur during the dynamic course of the game and are released behind the free throw line at approximately 9 m distance from the goal, usually have ball flight times of less than 435 ms that also impose high spatio-temporal pressure on handball goalkeepers (1, 2). As a result, goalkeepers frequently have to anticipate their opponent’s action and subsequently initiate their movement before definite ball flight information becomes available in both standardised (e.g., 7 m penalty) and less standardised (e.g., backcourt throw) situations to be at the right place at the right time to save the goal (3, 4). Thus far, research on anticipation in handball goalkeeping has mainly focussed on standardised 7 m throw situations, whereas research on less standardised situations such as backcourt throws is still sparse. This is remarkable in so far because backcourt throw situations occur more frequently and result in more goals than penalty situations (5). Hence, with backcourt throws being crucial and potentially game-deciding situations within handball, research and practice would substantially benefit from better understanding anticipation of backcourt throws in handball goalkeeping. Here, we took a first step in this endeavour by examining expert goalkeepers’ and coaches’ views on anticipation and cue utilisation facing backcourt throws in handball goalkeeping.

Research on anticipation in sports has generally found that skilled athletes show superior anticipation compared to less skilled athletes and that athletes can use a variety of information sources for action outcome prediction (6). These information sources can be divided into two broad categories: kinematic cues from an (opponent’s) movement pattern (e.g., from upper body, throwing arm, throwing hand (7)); and contextual cues from the domain, sport and situation-specific context an action is embedded in (e.g., action patterns, on-court position (8, 9, 10)). Currently debated models of skilled anticipation assume that information emanating from different sources could be integrated dynamically following a Bayesian strategy through continuously updated beliefs about probable action outcome (11). Reliance on contextual information is suggested to be more pronounced before or in the early stages of the opponent’s movement execution, when comparatively relatively little definite kinematic information is yet available, whereas reliance on kinematic information becomes increasingly dominant as the action unfolds (12, 13). The relative relevance and utilisation of different kinematic and contextual information sources for anticipation is likely to vary depending on differing spatio-temporal demands across domains (e.g., goalkeeping), sports (e.g., handball) and situations (e.g., backcourt throws) (4). That is, the kinematic and contextual cues assumed to inform anticipation of shot direction in, for example, soccer goalkeeping (14) can differ from those considered relevant for anticipation of throw direction in handball goalkeeping (7). Even within handball goalkeeping the kinematic and contextual information relevant to anticipation are likely to differ between situations such as 7 m throws and backcourt throws. More specifically, each situation has task-specific constraints that athletes are subjected to and that result in situation-specific kinematics and context-dependent action probabilities. For instance, whether defense players are present (which is usually the case in backcourt throws) or not (in 7 m throws) in a goalkeeper’s field of vision will influence which kinematic and contextual information are available and might potentially be used for anticipation. For that reason, anticipation and cue utilisation needs to be investigated within specific domains, sports and situations, such as backcourt throws in handball goalkeeping, to enable targeted and specific interpretation of findings and consequently also complement the general understanding of anticipation in sport.

In handball goalkeeping, a variety of kinematic and contextual cues have been shown to influence goalkeepers’ anticipation of throw outcome particularly in the situation of 7 m throws [for an overview, see (2)]. With regard to kinematic cue usage, for example, skilled but not novice goalkeepers are have been found to integrate information from multiple, globally distributed kinematic cues into anticipatory judgments (7). Moreover, anticipation seems to be more difficult against less frequently encountered left-handed than more familiar right-handed throws (15) irrespective of the observer’s handedness (16). The use of contextual cues in handball goalkeeping has been demonstrated using the example of opponent’s action preferences. Specifically, goalkeepers’ directional predictions of 7 m throws were more accurate if a thrower acted according to their directional preference, but goalkeepers’ anticipatory performance decreased against throwers who did not act according to their preference (17). This finding is underlined by other evidence within (18, 19) and outside the domain of goalkeeping [(20) in volleyball (21), in tennis], demonstrating that an opponent’s action preferences provide crucial contextual information for anticipation.

The majority of studies on anticipation in handball goalkeeping have been carried out in standardised 7 m throw situations while research on anticipation in less standardised situations such as backcourt throws is sparse [see only (22–24)]. Backcourt throws are by their very nature more complex compared to 7 m throws, as there are usually more players involved in the action and throws emanate from the dynamic course of a game. Importantly, they also occur more frequently during the game and result in a higher total number of goals compared to 7 m throws (e.g., 3,200 backcourt throws vs. 697 7 m throws at the World Championship 2015 (5)). Thus, considering goalkeepers’ important role for success in handball, investigating their cue usage in backcourt throw situations is important to broaden understanding of the processes underlying anticipation and to ultimately enhance anticipatory performance. In a first attempt to do so, Hatzl (22) conducted kinematic analyses of backcourt throws to identify relevant information sources for anticipation of throw outcome. Results showed that movement of the throwing hand and ball, hip and shoulder angle, distance between the ball and trunk as well as throwers’ relative shoulder width were suggested potentially relevant kinematic information sources for goalkeepers’ anticipation of backcourt throws. Even though this study provided a first glance on potentially relevant cues for backcourt throw anticipation, as well as their complexity and temporal availability during the unfolding action, the role of contextual cues was not addressed and the work has thus far only been followed up by three studies. First, Gutierrez-Davila, Rojas, Ortega, Campos and Parraga (25) found that handball goalkeepers initiate their defensive movement before backcourt throw release and that movement initiation is earlier under relatively certain compared to relatively uncertain throw conditions. Afterwards, Rojas et al. (24) found that experienced handball goalkeepers show superior anticipation compared to inexperienced handball goalkeepers and that they tend to initiate their movement later compared to their inexperienced counterparts. Finally, Huesmann et al. (23) generally confirmed the findings on expertise-dependent differences in anticipation of backcourt throws and also revealed that these differences were more pronounced under a perception-action simulated (video-based, movement response) compared to a perception-action artificial (video-based, key press) experimental condition. However, to the best of our knowledge there are no further studies that have investigated anticipation of backcourt throws as a highly complex and frequently occurring situation in handball goalkeeping.

Qualitative semi-structured interviews constitute a fruitful and holistic approach to depict the complexity of a (new) area of research and provide a structure and raise research questions for future quantitative and qualitative studies in the field [e.g., see (21, 26, 27)]. Using this approach, Schläppi-Lienhard et al. (26) for instance identified factors that influence decision-making in expert beach volleyball defence players (e.g., opponent specifics, opponents’ movements, situational context) and gained information on interactions between these factors. Connor, Renshaw and Farrow (28) interviewed cricket coaches and were able to establish that on a timeline from pre-ball to between-ball phases, different key factors such as prior knowledge on the opponents and own action capabilities, perception and constant updating of affordances and utilisation of contextual and kinematic information as well as action reflection define cricket batting performance. Finally, Morris-Binelli, van Rens, Muller and Rosalie (29) drew upon the rich knowledge of both expert field-hockey players and coaches to learn about anticipation of drag-flick penalty corners. Athletes and coaches reported that pre-match video analysis of attacking patterns and capabilities of the opposing team as well as in-game perception of kinematic and contextual cues like the attacker’s step width, stick angle or the opposing team’s positioning on field provide crucial information for anticipation. Additionally, psychological factors such as for instance resilience or leadership and communication were suggested to influence the usability of these cues. All of these studies resulted in thorough overviews on the situation-specific topics while also providing a basis for future research and informing practice about the complexity of information sources that are relevant for and may facilitate performance in the situation at hand. Arguably, research and practice in other domains, sports and situations may also benefit from the complex basis and structure a qualitative approach can provide for a (new) field of research.

The purpose of this study was to broaden the limited scope of research on anticipation of backcourt throws in handball goalkeeping. Specifically, we were interested in gaining a thorough understanding on the kinematic and contextual information sources for skilled anticipation of backcourt throws in handball goalkeeping to develop a comprehensive overview on the topic that (a) lays a basis for future quantitative and qualitative research in the field and (b) informs coaches’ and goalkeepers’ work on anticipation and cue utilisation in handball goalkeeping. To address our study aim, we followed the example of former qualitative approaches for an initial scope on anticipation and decision-making in sports (21, 26–29) by conducting semi-structured interviews with expert goalkeeper coaches and goalkeepers in handball.

## 2. Materials and methods

The study was approved by the local ethics committee at the University of Oldenburg (code: EK/2021/083). The study design considers the Eight “Big Tent” Criteria for Excellent Qualitative Research [(30), also see (31)] and reporting adheres to the Consolidated Criteria for Reporting Qualitative Research Guideline [COREQ (32)] as well as the APA Publications and Communications Board Task Force Report (33).

### 2.1. Participants

Six expert handball goalkeepers and goalkeeper coaches (*n = *2 female, *n = *4 male; *M*_age_ = 39.6; *SD = *8.8 years) from the First German Handball League and the German national team were recruited for the interview. Three participants were expert goalkeepers, two participants were expert goalkeepers and expert coaches and one participant had been an expert goalkeeper and was now an expert coach at the time of the interviews. To achieve rich rigour, both goalkeepers and goalkeeper coaches of different ages and experience, though all on an expert level, were included in the sample (for a similar approach, see (29) for more detailed information on the sample, see Table 1).

**Table 1:** Participant information.

	Experience as Goalkeeper		Experience as Goalkeeper Coach	
No.	Highest league	Years in highest league	Highest league	Years in highest league
1	1st league	22	1st league	2
2	1st league	19	1st league	5
3	1st league	4	–	–
4	1st league	20	–	–
5	1st league*	18	National team	3
6	1st league	2	–	–

* Retired.

All participants also either played or still play as a goalkeeper on a national team.

Recruitment was coordinated in cooperation with the German Handball Federation and potential participants were contacted via e-mail. Participants were recruited until data saturation was reached and this was based upon the degree to which information mentioned in the previous interviews was repeated in new data collections (34). Since we did not identify new information in the final two interviews that had not been mentioned in the first four interviews, we completed data collection after a total of six interviews. Even though it needs to be acknowledged that we cannot rule out that further data collections would have yielded more information, we feel confident to have reached a high degree of data saturation by recruiting a broad sample of athletes with high expertise and following the conventions for data saturation. All participants provided written informed consent prior to the interviews.

### 2.2. Researchers

The interviews were conducted by two researchers from the group. The first researcher is the female author KH (26 years old) who obtains a Master of Education degree and works as a researcher and PhD student in sport science. KH’s research focusses on anticipation, perception(-action), expertise and cognition. The second researcher is the male co-author JW, who was, at the time of data collection, a Master of Education candidate (24 years old). Additionally, JW worked as a student research assistant with a focus on anticipation and decision-making in sports. As indicated by their fields of research, the interviewers obtained prior knowledge on anticipation in sports which allowed them to establish and guide professional conversation as well as understand participants’ responses. Further, suggestive questions were consciously avoided to ensure that the researchers’ understanding on the topic would not influence participants’ responses. The interviewers conducted two pilot interviews before data collection to prepare for and familiarise themselves with the technical equipment and interview guide. During the interviews, KH was the interviewer and JW was responsible for the software (e.g., audio recording). The researchers and participants established no relationship except for e-mail recruitment prior to the interviews. Therefore, participants were only familiar with the researchers’ names and institution as well as the general aim to conduct interviews on anticipation in handball goalkeeping.

### 2.3. Interviews

The authors developed a semi-structured interview guide prior to data collection that was piloted with two volunteers who were not part of the sample. The interview guide ensured that all participants were asked the same questions and also allowed for non-scripted follow-up questions to further explore responses of the participants [(35); for the interview guide, see Table 2]. Question 1 (Q1) served the purpose to familiarise participants with the interview situation by having them talk openly about a variety of skills that are crucial for successful handball goalkeeping. Q2 then shifted the participants’ focus towards backcourt throws and was followed by Q3 which specifically targeted anticipation of backcourt throws. Q4 aimed to identify kinematic information sources that participants find important for anticipation of backcourt throw outcome and was followed up by a more in-depth question on how these kinematic information sources influence anticipation in Q.4.1. Q5 then aimed to identify contextual information sources for anticipation of backcourt throw outcome and was also followed up by a more in-depth question on how these contextual information sources influence anticipation in Q5.1. Q4 to Q5.1 are the questions that most specifically targeted this study’s research question. We consciously chose not to use the scientific terms *kinematic* and *contextual information* in the questions to avoid the possibility that different concepts of these terms might influence participants’ responses. Finally, Q6 served to cover aspects that the participants felt had not been discussed in the interview.^1^ The interviewers were free to ask non-scripted follow-up questions throughout the semi-structured interviews (35). These follow-up questions mostly concerned specific links between information sources and potential action outcomes and provided increased richness of the data.

**Table 2:** Interview guide.

No.	Question
1	What skills are crucial for a goalkeeper to be successful at the highest level?
2	Please imagine a situation where a player executes a backcourt throw at the goal. Describe what the goalkeeper pays or has to pay attention to in order to save the goal.
3	Please elaborate how important you consider anticipation of throw direction here.
4	What information from an opponent’s movement do you consider important for anticipation of backcourt throw direction?
4.1	Please elaborate how this movement information helps goalkeepers anticipate backcourt throw direction.
5	What other information, besides movement information, do you consider important for anticipation of backcourt throw direction?
5.1	Please elaborate how these information sources help goalkeepers anticipate backcourt throw direction.
6	Is there anything you would like to add to what we have covered in this interview?

### 2.4. Procedure

First, potential participants were contacted and provided general information on the study. Afterwards, interviews with participants were scheduled for a date and time that suited both participants and researchers. Participants were then sent a link to a survey that was created in LimeSurvey (www.limesurvey.org). The survey contained general information on the study and the informed consent sheet which they were asked to read and, if they wished to take part in the study, virtually sign. They were then forwarded to a pre-interview questionnaire on personal data such as their age, playing position and playing and/or coaching league which was used to describe the sample. Participants were free to fill out the survey at any time prior to the interviews.

The semi-structured interviews were conducted online via Skype^TM^ (www.skype.com). The reason for this was that for one, participants played or coached handball all over Germany and in-person data collection would have been highly time-consuming, costly and environmentally unfriendly and further, the situation of the COVID-19 pandemic did not allow for reliable planning of in-person data collection. The interviewers each used DELL Latitude 5510 laptops and participants used their personal device of choice for the interview. Audio was recorded using Skype^TM^ as well as the open source software Audacity (www.audacityteam.org) for backup. First, the researchers and participants introduced themselves and any questions regarding the interview procedure were addressed. Then, the interviews followed the earlier introduced interview guide. The transcribed interview recordings were between 13:53 min and 25:47 min long (*M =* 20:21 min, *SD* = 4:29 min). These durations refer to conversation on the interview topic and exclude the introduction, questions regarding the interview procedure and farewell. Importantly, the length of the interviews did not result in differences of information sources mentioned but rather influenced the depth with which information sources could be elaborated on and the participants’ ability to explicitly link information sources to action outcome probabilities. Coaches tended to display more explicit, declarative knowledge resulting in longer interview durations, potentially due to the fact that it is a crucial part of a coach’s job to verbalize knowledge on their sport.

### 2.5. Data analysis

The data analysis was carried out using a thematic analysis following the six-step guide proposed by Braun and colleagues (36, 37). The interviews were transcribed verbatim using the software sonix (sonix.ai). Transcriptions were revised and corrected on the basis of the audio recordings. In phase 1 of the thematic analysis, KH and JW both familiarised themselves with the data by repeatedly and analytically reading the transcripts for understanding. In phase 2, they independently and manually coded the material with regard to the study’s underlying aim. That is, the researchers marked all chunks of text that included information on sources that participants named as relevant for anticipation of backcourt throw outcome in handball goalkeeping and collapsed this information into codes. During this process, KH and JW went over the material several times, revisiting and critically evaluating their coding until they felt confident that all information from the interviews was accurately represented in the codes. Afterwards, KH and JW served as each other’s critical friend which involved comparing the individually identified codes and discussing themes. In the seldom case when KH and JW’s coding differed, they reviewed the participant’s response and were able to establish consensus based on the interview material. In phases 3, 4 and 5, KH and JW as well as the authors JS and FL organised the codes into themes, repeatedly reviewed these themes, defined each of them and finally settled on a total of three overarching themes with multiple sub-themes each. More specifically, they agreed upon the ‘type of information source’ as one overarching theme, including the deductive, well-established differentiation between ‘kinematic’ and ‘contextual’ information (4) and the inductive ‘influential factors for cue availability and utilisation’ as sub-themes. Further, they inductively identified a differentiation for ‘agent’ with sub-themes ‘offence’ and ‘defence’, containing the second sub-themes ‘thrower’ and ‘opposing team’ as well as ‘defensive players’, ‘block’ and ‘goalkeeper’. Finally, they found ‘temporal availability of information source’ as a third overarching theme, including the sub-themes ‘before game’, ‘before throw’ and ‘during throw’. In an additional step to increase rigour, we conducted member reflections in which this primary classification including themes, sub-themes, second sub-themes and codes, was provided to the participants. They were then asked to provide general feedback on the classification and indicate whether they felt like any relevant information sources were missing. None of the participants wanted to add aspects, however, one participant noted that the list of information sources was generally very complex. After the member reflections, the data analysis was finalised and phase 6, the production of the report, commenced (36–38). The structure of the results section will follow the overarching theme ‘type of information source’ with its sub-themes ‘kinematic information’, ‘contextual information’ and ‘influential factors for cue availability and utilisation’. The structure within these sections will then follow the overarching themes ‘agent’ and ‘temporal availability of the information source’ as well as their sub-themes and second sub-themes to facilitate readability.

## 3. Results

Participants emphasised the “short time window in which the ball is thrown” (P1), that is, the high spatio-temporal pressure a handball goalkeeper is exposed to and the role of “anticipation as a fundamental skill” (P4) for successful interception of backcourt throws. Here, participants named a variety of information sources that influence anticipation of backcourt throw outcome. They emphasised that information sources for anticipation can be acquired during games, through the coaches and in video analyses.

**Figure 1:**
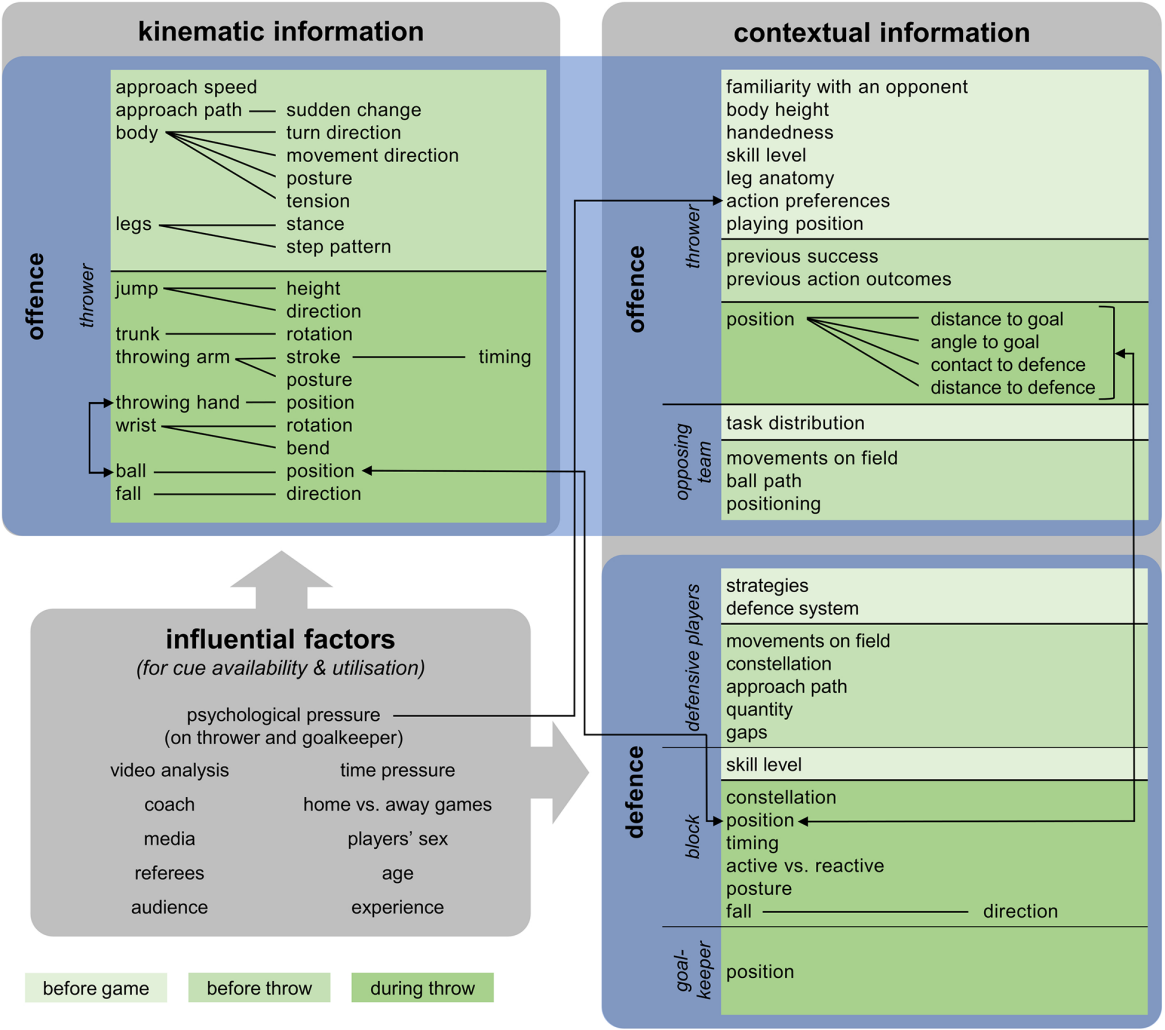
Cues considered relevant by interviewees for anticipation of backcourt throws in handball goalkeeping. Information sources found within the same box divided by a black line show the mentioned information source on the left and the mentioned specification(s) on the right end of the line [e.g., approach path—sudden change (in approach path)]. Black arrows indicate interactions between information sources (e.g., ball position in relation to block position).

Generally, the information sources that were considered explicitly relevant for anticipation of backcourt throws can be defined by the three complementary, overarching themes introduced above: ‘type of information source’, ‘agent’ and ‘temporal availability of the information source’. The ‘type of information source’ can either be ‘kinematic’, ‘contextual’ or an ‘influential factor for cue availability and utilisation’, whereas the ‘agent’ can be the ‘offence’, and more specifically the ‘thrower’ or the ‘opposing team’ or the ‘defence’ and more specifically the ‘defensive players’, the ‘block’ or the ‘goalkeeper’. Finally, temporal availability of the information source can occur ‘before the game’, ‘before the throw’ or ‘during the throw’. Importantly, some but not all of the mentioned cues were linked to specific throw outcome probabilities. Figure 1 provides an overview of all identified information sources.

### 3.1. Kinematic information

Participants stated that they use information from the *thrower’s* movement for anticipation of backcourt throw outcome that is available before or during the throw. *Before the throw*, the thrower’s approach speed as well as approach path and sudden changes in the approach path appear crucial for throw prediction. Participants mentioned for example that “if the run-up is straight, they [the throwers] throw straight. If they approach diagonally through the middle of the court after crossing or so, they throw diagonally” (P3). Further, movement information such as turn direction, general movement direction, posture and body tension as well as the thrower’s legs, their step pattern and stance are considered relevant for anticipation of backcourt throw outcome.

*During the throwing action*, goalkeepers and coaches consider “[…] where the player jumps. Because if a player jumps straight at the defence player, they will almost always throw straight past them” (P3) and “nevertheless, of course, […] also jump height determines what a thrower can do” (P2). They also stated that throwers’ body rotation provides information on throw outcome and influences anticipation, elaborating that “if you want to generalise this, a lot of rotation in the upper body means it’s likely a diagonal throw” (P4). Participants further highlighted the importance of end-effector information from the throwing arm, the throwing hand, wrist and ball for anticipation of backcourt throws. For the throwing arm, goalkeepers consider posture, stroke and stroke timing: “How is the player holding their arm? Will they throw high or low around the defence?” (P4). Further, perception of the throwing arm timing which is complemented by information on throwing hand position as well as wrist bend and wrist rotation plays a role in anticipation. Here, P1 stated that “the wrist shows that the thrower will throw towards [a certain direction]. Because if you would try to execute this action and throw towards the other corner, you would almost break your wrist.” Responses also underline how crucial experts consider the ball, and more specifically ball position and its interaction with the throwing hand for anticipation of backcourt throw outcome: “Above all, I have to keep my eyes on the ball […] and if you manage to focus on the ball […] and on everything else peripherally, that’s great” (P4). They further elaborated that “[…] when a young goalkeeper asks me [what to focus on], I always say you have to learn and practice to focus on the ball and really perceive how it’s laying in the [throwers’] hand, to know how it can be thrown, where it can be thrown past.” Finally, participants noted that they consider kinematic cues on the thrower’s fall and fall direction late during the throw. Here, information on whether “the player falls into [the defence] or somehow [falls] before that due to contact” (P4) can influence the trajectory the ball can take or the position at which the ball can be thrown off and therefore is considered explicitly relevant for the prediction of backcourt throw outcome in handball goalkeeping.

### 3.2. Contextual information

Goalkeepers and coaches considered several contextual information sources from the offence, that is the thrower, the opposing team and the defence, made up of the defensive players, the block and the goalkeeper themself as explicitly relevant for anticipation of backcourt throws. These information sources become available to the goalkeeper either before the game, before the throw or during the throw. *Before the game*, goalkeepers know whom they are playing against and participants report to use familiarity with a *thrower* to anticipate their actions. Familiarity with an opponent can be acquired in previous encounters, through coaching or video analyses and can provide a variety of further cues, such as an opponent’s action preferences: “There are players who always like to throw the difficult shot. Even in backcourt throws, they […] always do the difficult shot. And if you know that and can anticipate something like that, […] it becomes easier” (P3). Further, participants mentioned that they also use prior knowledge on the thrower’s body height, their handedness, leg anatomy, and playing position for anticipation of backcourt throws. Before the actual throw, but already *during the game*, previous action outcomes as well as previous success might provide crucial information on where the thrower is likely to place the backcourt throw: “How did the game go so far? What kind of throws did the thrower execute before? Successful, unsuccessful?” (P5). Goalkeepers and coaches mentioned that during the throw, the thrower’s on-court position plays an important role for anticipation of backcourt throw outcome. In particular, the distance and the angle between the thrower and the goal were considered crucial “[…] because from certain angles, it might not be possible to throw the ball towards a certain corner at all […] and so I don’t have to cover that corner at that moment” (P4) as well as contact to defence and distance to defence.

Participants mentioned that *before the game* knowledge on task distributions within the *opposing team* can be used to anticipate who will likely execute throws on goal: “Who are the scorers? Who does the team want to bring into play? Who is taking responsibility?” Interestingly, here, usage of task distribution seems to be at least partially dependent on game situation: “Especially in a tight situation, the player [who is taking responsibility] will probably take the lead […] and you can focus on that already” (P2). *Before the throw*, participants rely on movements on field, ball path as well as player positioning as contextual cues for anticipation. For instance, they ask themselves: “What are the movements on field?” (P5), “Where does the ball come from?” (P3) and they state that “[…] the position of the player who throws the ball to the player who executes the throw on the goal also has an influence because it decides the ball path and you can just observe [the previous thrower’s] arm flow and you know [which shot] might be easy or difficult for the opponent” (P1).

The aforementioned information sources focussed on information from the offence, however, goalkeepers and coaches note that information from the defensive players, the block specifically, as well as the goalkeeper themself can influence anticipation of backcourt throws. *Before a game*, the coach, the goalkeeper and the field players discuss the *defensive players*, and more specifically the defence system as well as defence strategies which can help the goalkeeper to anticipate: “Of course, we often have clear agreements on which side [of the court] we want to allow the opponents to throw from and which not. That’s a very, very important source of information for me, if not perhaps the most important overall, because I always pay attention to what the defence does […]” (P4). This early contextual information is further complemented *before the throw* by the constellation of players who are part of the defence, their movements on field and approach path towards their opponents, as well as quantity of and gaps in the defence ultimately.

From the *block* specifically, interviewees reported that they use *before game* knowledge on the blocking players’ skill level to anticipate how they will perform in the defence and adapt their anticipation of throw outcome accordingly. Participants further highlighted that *during the throw*, block constellation, that is, how many players and who are blocking the throw, delivers crucial cues regarding the thrower’s action outcome. They elaborated that “a double block is unfortunately very unrealistic because it happens very, very rarely.” (P5) but also that “if there is a double block, then there is even less space [to throw the ball]. Then I usually don’t even see the thrower anymore, so it’s really just a matter of being patient and seeing where the ball is likely to pass at the last moment […] but actually, ideally, I try to position myself so that I can see the thrower somehow. But of course, that can be difficult when a double block suddenly occurs” (P6). Responses that expressed the importance of block constellation were often accompanied by remarks on block position. For instance, participants mentioned that they consider “[…] whether the block is in position” and that “if a player attempts to throw past the block, I always anticipate the straight shot” (P3), as “of course, it is more difficult to throw the ball around the block player” (P1). Besides block position, these responses also show the importance of the interaction between the thrower’s position and the block position as well as the block timing for anticipation of backcourt throw outcome. Participants mentioned that they further consider block position in relation to ball position during the throw as this interaction can provide information on whether, and if so, where the ball will likely be thrown past the block. Additionally, respondents reported to utilise information on whether a block is active or reactive, that is, whether the blocking players actively attack the thrower in order to directly impair the throw or whether they let the thrower execute the throw but try to close any potential throw paths. Lastly, posture of the blocking players and blocking players’ fall as well as specifically the block’s fall direction during the throw were also considered crucial cues to anticipate the thrower’s action during backcourt throws.

Participants also mentioned that *during the throw*, “through my own positioning, I try to tempt the opponent to throw towards a certain corner” (P1). Evidently, goalkeepers seem to manipulate their own position within the goal to deceive the opponent and then use this information for anticipation. For instance, they might intentionally leave a corner free and anticipate that the thrower will notice the free corner and direct their throw towards there. However, potentially unbeknownst to the thrower, the goalkeeper leaves the corner free on purpose and already anticipates that the thrower will make use of this apparent advantage and throw towards the free corner, leaving the goalkeeper with sufficient time to execute an action towards the free corner of the goal.

### 3.3. Influential factors for cue availability and utilisation

Finally, a few rather unspecific influential factors were mentioned as explicitly relevant for cue availability and utilisation in anticipation of backcourt throw outcome. These factors do not inform about action outcome like, as stated above, a high degree of body rotation would suggest a diagonal throw or knowing that an opponent likes to throw to the top right corner would increase the odds of the throw going towards that corner. However, they are suggested to influence which kinematic and contextual information sources are likely to be used during the unfolding action and therefore may play a crucial role in anticipation of backcourt throws.

First, participants frequently emphasised the importance of video analysis to scout the upcoming opponent: “I watch a lot of videos [of our opponents] actually. […] you still watch videos of the last two or three games they played, even if you have already played against them many times […] and I even used to differentiate between home and away games” (P1). Video analysis generally increases familiarity with an opponent and can include information on an opponent’s kinematics, action preferences, the opposing team’s task distributions and a variety of other cues that goalkeepers ultimately report to use for anticipation of backcourt throw outcome. Even differences between home and away games, as mentioned by P1, might be a factor that is considered during video analysis as well as during the game. Further, responses show that athletes’ sex potentially influences utilisation of different information sources. For instance, it was mentioned that “men’s handball is faster [compared to women’s handball]. They’ll sometimes say ‘I take this corner and the defence takes that corner.’ In women’s handball, there is a relatively good chance that you can anticipate [based on kinematics] and position yourself correctly” (P2). Additionally, the goalkeeper’s age and experience, the coach, referee, media, audience, time pressure as well as psychological pressure on the thrower and the goalkeeper were considered influential for cue availability and utilisation in anticipation of backcourt throw outcome. For example, participants indicated that depending on their age and experience, goalkeepers might or might not be able to use certain crucial information sources for anticipation: “[…] the junior goalkeepers might not yet master the interplay with the defence” (P1), and therefore, utilisation of information from the defence for anticipation could at the least be impaired. Participants also stated that “[…] under pressure, people always act intuitively. […] and if I remember their favourite throws, that will help me immensely during the last five minutes of a close game” (P3). Evidently, it appears that knowledge of psychological pressure on the thrower can influence how goalkeepers weigh certain information sources for anticipation of action outcome. However, on the other hand, psychological pressure on the goalkeeper was also mentioned to influence their general ability to anticipate as well as utilisation of kinematic and contextual information sources according to the respondents.

## 4. Discussion

The aim of this study was to determine the information sources expert handball goalkeepers and coaches consider explicitly relevant for anticipation of backcourt throws. Generally, participants’ responses portray the complexity of information sources that influence anticipatory judgement. Results reveal numerous kinematic information sources from the thrower that become available before or during the throw as well as contextual information sources from the thrower, the opposing team, the defensive players, the block and the goalkeeper themself that become available before the game, before or during the throw as relevant for anticipation of backcourt throw outcome. Some information sources, such as thrower position and block position, seem to interact and jointly provide goalkeepers with cues on backcourt throw outcome. Additionally, some influential factors such as video analysis or psychological pressure on the thrower and the goalkeeper might provide a variety of different information sources and affect availability and utilisation for anticipation of backcourt throws. These classifications allowed for an overview of information guiding anticipation of backcourt throws in handball that includes the information sources further defined by the type information source, agent, temporal availability as well as representation of interactions (Figure 1).

Responses generally confirmed and substantially extended the currently limited state of research on the topic by providing a variety of mostly novel information sources relevant for anticipation of backcourt throws in handball goalkeeping. Earlier findings by Hatzl (22) which showed that movement direction of the ball and hand, upper body rotation as well as ball position are crucial kinematic cues for anticipation of backcourt throws were affirmed by the results in this study. Additionally, a variety of information sources formerly identified in other sports or situations, such as upper body, throwing arm, hand and ball [in handball 7 m throws (7)] legs and trunk [in soccer penalties (19)], opponents’ action preferences [in handball 7 m throws (17)] as well knowledge from pre-match video analysis containing a multitude of information sources [in field hockey drag-flicks (29)], to name only a few, appear to have an influence on anticipation of backcourt throws in handball as well. Conversely, some information sources which have been identified as relevant for anticipation in other sports and situations [e.g., hips in soccer penalties (19)] were not explicitly mentioned in this study while other information sources, for example, the thrower’s approach speed or block constellation are, to the best of our knowledge, explicitly identified as relevant for anticipation for the first time not only in anticipation of backcourt throws but in research on anticipation in sports in general. These findings could therefore also spark research in other sports and further substantiate the assumption of domain, sport and task-specificity of certain information sources for anticipation in sport.

Evidently, the complexity of information sources identified as explicitly relevant for anticipation of backcourt throws and the limits of (implicit) information-processing capacity suggest that not all cues can be used for anticipation of every backcourt throw (39). Conveniently, by usually limiting information pick up to processable amounts of information, goalkeepers are likely to primarily process information they consider relevant for successful task completion (40). This can, for instance, be influenced by former experience in similar situations or training where goalkeepers either explicitly or implicitly developed knowledge bases on information-rich cues and their likely effect on action outcome (41, 42). Therefore, even if a variety of information sources appears explicitly relevant for anticipation, probably only a selection of them are used in the actual situation and this selection might further vary depending on several factors discussed below. Future experimental work would need to assess their actual role for and integration in handball goalkeepers’ anticipation of backcourt throws under experimental conditions that allow for targeted investigation of specific information sources while considering representative designs that allow for transfer to the field (43).

Besides boundaries of information processing ability, cue utilisation might be further influenced by situation-specific parameters. First, reliance on a specific cue is constrained by general availability of this information source. For instance, an opponent’s action preferences can only be used for anticipation if they are known and wrist bend can only influence anticipation if it is visible to the goalkeeper (i.e., vision is not blocked by e.g., field players). Second, our results suggest that temporal availability of information sources plays an important role in the utilisation of cues for anticipation [also see (28)]. Research on the topic generally indicates that contextual information usually is or becomes available earlier compared to kinematic information and is therefore relied on more heavily during early phases of the action, whereas kinematic information becomes increasingly important during the late stages of action execution (4, 12, 44). Third, other situational constraints, such as the audience and media, psychological pressure as well as the players’ sex were mentioned to influence cue utilisation. For instance, the audience or score might induce psychological pressure on the goalkeeper or thrower which then might lead to generally impaired anticipation (45) or, as mentioned in the interviews, to a heavier focus on specific information sources such as action preferences. Furthermore, participants mentioned that differences in temporal pressure between men’s and women’s handball can influence cue utilisation in the way that male goalkeepers may need to rely heavier on earlier contextual information, whereas female goalkeepers might be able to wait for potentially more reliable kinematic information and still be able to successfully save the goal.

Although the pool of relevant information sources for anticipation is usually narrowed down by the aforementioned constraints, goalkeepers still have to weigh available cues and ultimately decide where to move in order to intercept the ball. In the last few decades, a variety of models have been developed to explain how different kinematic and contextual cues are integrated into anticipatory judgements (6). Recent research for instance suggests that information processing and subsequent action prediction might follow a Bayesian strategy [e.g., see (11)]. More specifically, athletes might form predictions based on early available cues, such as previous action outcomes, which are then subsequently and continuously updated based on sensory input that may provide additional anticipation-relevant cues. These cues can either confirm and thus strengthen the earlier prediction or dismiss and thus weaken it, which ultimately leads to an action prediction that is based upon action probabilities accumulated from various contextual and kinematic information sources (44, 46–49). Following these suggestions, goalkeepers might actually be able to integrate a variety of the identified information sources into their judgement by considering them at different times and updating their prediction while the action unfolds. Here, specifically the classification of the identified cues regarding their temporal availability in this study provides a basis for future research regarding the question of computational integration (11, 12, 49). Moreover, it is necessary to investigate whether there is a general prioritization of certain information sources compared to others which might, as discussed before, be mediated by situational constraints as well as inter- and intra-individual differences.

Another factor that might influence cue utilisation for anticipation of backcourt throws in handball are interactions between information sources. Participants indicated that throwing hand position and ball position, ball position and block position, thrower position and block position as well as action preferences and psychological pressure on athletes form interactions that portray crucial information for anticipation. Our study findings suggest that these interactions can take on different forms, that is, some of them carry information within the relation between action outcome cues (e.g., hand position relative to ball position defines action possibilities), while others influence utilisation of one cue in the presence of another cue or circumstantial factors (e.g., psychological pressure increases reliance on action preferences). Importantly, such interactions as well as their utilisation for anticipation could very well vary inter- and intra-individually [e.g., (50, 51)], which might be especially pronounced when interactions involve circumstantial factors such as psychological pressure (45). However, in order to gain a thorough understanding of interactions between information sources and their utilisation for anticipation, a rather holistic research approach is needed that combines several information sources and ideally incorporates perception-action coupled experimental methods to allow for conclusions on transfer to the field.

Finally, a few methodological study limitations need to be acknowledged. For one, the complexity of information sources that were considered explicitly important for anticipation of backcourt throw outcome restricted the depth with which each cue could be discussed. For that reason, we were unable to explore specific links of information sources and action outcome probabilities for all cues. This was even further influenced by the fact that participants were often unable to provide specific links between cues and action outcome probabilities, possibly due to lack of declarative knowledge on the matter. Further, we exploratively analysed the responses with regard to potential differences between female and male as well as goalkeeper and coach participants. Tentatively, coaches seem to obtain a greater explicit and declarative knowledge base on anticipation of backcourt throws as indicated by the extent of their responses. This could be explained by the inherent tasks that come with being a coach which requires them to frequently verbalize their declarative knowledge to instruct or give feedback to their athletes, whereas athletes tend to be the ones executing an action without the need to verbalize it. Furthermore, coaches, especially in high leagues, usually have played their sport on a high performance level for a long time and therefore obtain extensive sport-specific experience themselves. However, such comparisons were not the aim of this study and are only cautiously introduced due to the limited number of participants for each group.

In conclusion, this study portrays the complexity of information sources that expert handball goalkeepers and goalkeeper coaches consider explicitly relevant for anticipation of backcourt throws. It provides a basis for a needed extension of research on anticipation in this complex sport-specific situation in handball, which is the most frequent situation goalkeepers encounter during matches (5). From an applied perspective, handball goalkeepers and coaches might use these results as a basis for their work on cue utilisation and to explore potential links between selected information sources and action outcome probabilities. Here, it is especially crucial that coaches acknowledge the complexity of information sources goalkeepers are confronted with and can potentially use for anticipation and utilise this complexity to focus on individual cue utilisation and training with their goalkeepers. Future studies should aim to experimentally verify if these information sources are crucial for anticipation of backcourt throws in laboratory settings as well as on-field, how they are weighted and ultimately integrated into anticipatory judgements, potential interactions between information sources, intra- and interindividual differences as well as training of backcourt throw anticipation [also see (52)]. Each following study will take a step towards the answers to these questions which can ultimately enhance our understanding of anticipation as a crucial aspect of sports performance to guide future research and practice.

## Data Availability

The datasets presented in this article are not readily available because of the data privacy statement agreed upon with participants. Anonymous excerpts of the transcript can be provided upon reasonable request. Requests to access the datasets should be directed to kim.jana.huesmann@uni-oldenburg.de.

## Footnotes

^1^Please note that after Q5, participants were further asked to (1) name their top 3 information sources handball goalkeepers should use for anticipation of backcourt throws and (2) name their top 3 information sources youth handball goalkeepers should use for anticipation of backcourt throws. We chose not to report the responses to these questions as the interviewees mostly mentioned less or more than three information sources and were unable to put them into order. This resulted in unclear findings for the top 3 information sources and a likely incomplete picture on information sources for anticipation of backcourt throws in youth handball goalkeeping which further overlapped with the responses to Q4 to Q5.1.
